# Martian buildings: structural forms using in-place sources

**DOI:** 10.1038/s41598-022-25507-5

**Published:** 2022-12-20

**Authors:** Omid Karimzade Soureshjani, Ali Massumi

**Affiliations:** Department of Civil Engineering, Faculty of Engineering, Kharazmi University, Tehran, Iran

**Keywords:** Astronomy and planetary science, Engineering

## Abstract

On Mars, structural loads and the low tensile strength of in-place Martian binders make existing solutions for Martian structures uneconomical because they are based on the terrestrial sources like inflatable units. Here we address this issue by introducing and analyzing three innovative structural forms in accordance with the structural engineering point of view using symmetric optimum parabolic rotated arch shapes and in-place waterless sulfur-based concrete. These forms minimize the tensile stresses under Martian structural loads. Probable Martian structural loads, including gravity, wind, marsquakes, asteroid and meteoroid impact loads and their effects have been investigated and calculated. The proposed models were analyzed under Martian structural loads using the implicit finite element method and the results were compared to two concrete structural forms from previous studies. The proposed models could tolerate Martian structural loads with complete elastic behavior and would significantly decrease the Martian colonization cost due to using Martian resources and reduce element importing from Earth.

## Introduction

Because of the economic opportunities, discovery of a new world, new energy sources and preserve our species, multiplanetary life is of interest^1,2^. Due to these reasons, multiplanetary life has received a lot of attention since the beginning of the twenty-first century. Also, with the increasing demand for energy (beyond the capacity of the Earth) in parallel with the passing of type 1 Kardashev scale, multi-planetary life seems inevitable^3,4^.

Some ideas have been proposed for terraforming Mars but there is a long way to go with this notion^5^. The Martian colonization solutions should be appropriate with today’s Martian harsh environment. Environmental factors affecting structural loads such as atmospheric pressure, marsquakes, wind, and asteroid and meteoroid impacts can be calculated using data from landers and orbiters such as InSight and Odyssey^6–9^. NASA has studied suitable structural forms for human settlement on Mars for a total mission time of 919 days^10^. The envisioned structural form is a cylindrical metal shape with a height and diameter of 8 m that is able to tolerate the difference between the internal and external pressure. However, shuttles (~ 110 to 120 tons) would carry these habitats (~ 50 tons) from Earth, increasing the costs dramatically, including vehicle development, fuel, management, increasing the number of shuttle launch and so on.

It is not feasible to transport all the structures required to create a settlement on Mars from Earth. However, because of the low tensile strength of Martian binders, building Martian structures with these in-place binders would be very challenging. The design of an optimum Martian base on the regolith using inflatable and tensile resistance materials has been assessed for covered and uncovered structural elements^11^. The design of a human-friendly Martian base is envisioned to consist of five structural units based on the terrestrial sources for structures by Kozicki and Kozicka^12^, who proposed the suitable design, size and area for the intended structures.

The structural components for creation of an outpost using a 3D printer were investigated using inflatable structural elements (as the internal unit) and the regolith as the binder (for external unit) ^13^. In this regard, structures below and near the surface are options but would require remarkable energy for construction^14,15^. It is possible to produce a durable, fast curing and stable concrete with high compressive strength (more than 100 MPa) using the Martian regolith. Sulfur-based concrete is one of the best options for use in a 3D structural printer^16,17^. A hybrid dome shape printable Martian structure that consists of a thin inflatable shell and printed regolith was proposed by Troemner et al.^18^. This structure that was a part of NASA’s habitat challenge could tolerate Martian structural loads as well as environmental loads.

In this paper the effect of the gravity load, difference in the interior and exterior pressures, wind loads, and marsquake loads were studied and calculated using data from Viking 1 and 2 and the InSight mission SEIS data to develop a design load map with which to design a Martian structure. The effects of asteroid and meteoroid impact loads also were assessed. Three innovative structural forms for a Martian settlement are proposed that use symmetric rotated optimum parabolic arches. These forms have been designed to minimize the tensile stress and maximize the compressive stress under Martian structural loading to meet the requirements of Martian concrete and eliminate the import of structural elements from Earth. The models were analyzed under Martian structural loading using the implicit finite element model (FEM) and the structural behavior of the models was compared with concrete structural forms from the studies of Cesaretti et al.^13^ and Kozicki and Kozicka^12^. The amount of concrete required for the construction of the models was investigated to determine which model would require the least energy to build.

## Martian structural loads

### Gravity load and internal pressure

Because Mars experiences less gravity acceleration than Earth, the self-weight of the Martian structural system would be much less than a terrestrial structure. The habitats should be designed to tolerate the 1 atm pressure difference between interior and exterior to satisfy human comfort. In addition to the structural self-weight, 1 atm of pressure would be applied as a constant distributed load inside the habitat.

### Wind load

**Figure 1:**
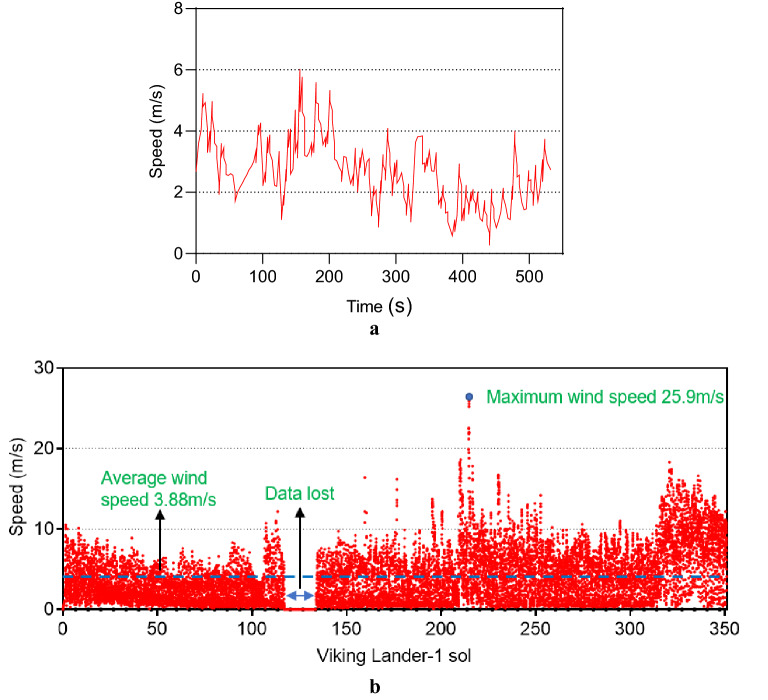
Martian wind speed recorded by Viking 1. **(a)** 9-min speed on sol 22. (**b)** Over the first 350 sols in each almost 1-h bin.

Maximum wind speeds of 9.5 m/s have been recorded by Viking 1 and 7.1 m/s by Viking 2 during the first 44 and 50 solar days (sols), respectively^9^. Figure 1a shows the speed recorded on sol 22 (9 min) by Viking 1^19^. As on Earth, the wind speed on Mars varies according to the season. For better accuracy, a longer time span should be considered to determine the wind speed. Figure 1b shows the wind speed in the first 350 sols (about half of a Martian year) recorded by Viking 1^19^. The maximum wind speed occurred on sol 214 with a speed of 25.9 m/s. The InSight wind speed observations during the first 220 sols showed a maximum wind speed of almost 25 m/s^20^, confirming good agreement between the Viking 1 and InSight wind speed observations.

The wind stagnation, velocity pressure and force for the maximum recorded wind speed of 25.9 m/s (93.24 km/h) were calculated using Eqs. (1–3) as being 411.20 N/m^2^, 342.52 N/m^2^, and 5,9917.02 N, respectively^21^. Wind is a fundamental atmospheric quantity caused by air movement. The wind force or pressure depends on the air density (ρ) or its mass per volume. At the surface at an average temperature, Mars air density is about 0.016 that of the Earth, which is 1.22 kg/m^3^ at 15 °C. The fluid dynamic pressure or wind stagnation pressure at the envisioned speed considering Mars air density was calculated as 6.70 N/m^2^ (Eq. 4). This suggests that, on Mars for a usual structure, the wind pressure or force would be 1.62% of that on Earth, which is almost 0.04% of the total Martian weight of proposed Model-1.

### Marsquake loads

During the 640 h mission of Viking 2, high-quality seismic data recorded in ambient silence, detected a possible marsquake at sol 80 with a probable magnitude of 3 on the Richter scale with a high-frequency content^7^. The seismometer was capable of detecting seismic motion of magnitude of 5 within an 0.021 area (1000 km) of the red planet. Up to September 2019, the InSight lander recorded 174 events, including 24 deep events (mantle origin) of a relatively high magnitude and low frequency content^22^. No marsquake with a magnitude greater than 3.8 Richter was recorded.

**Figure 2:**
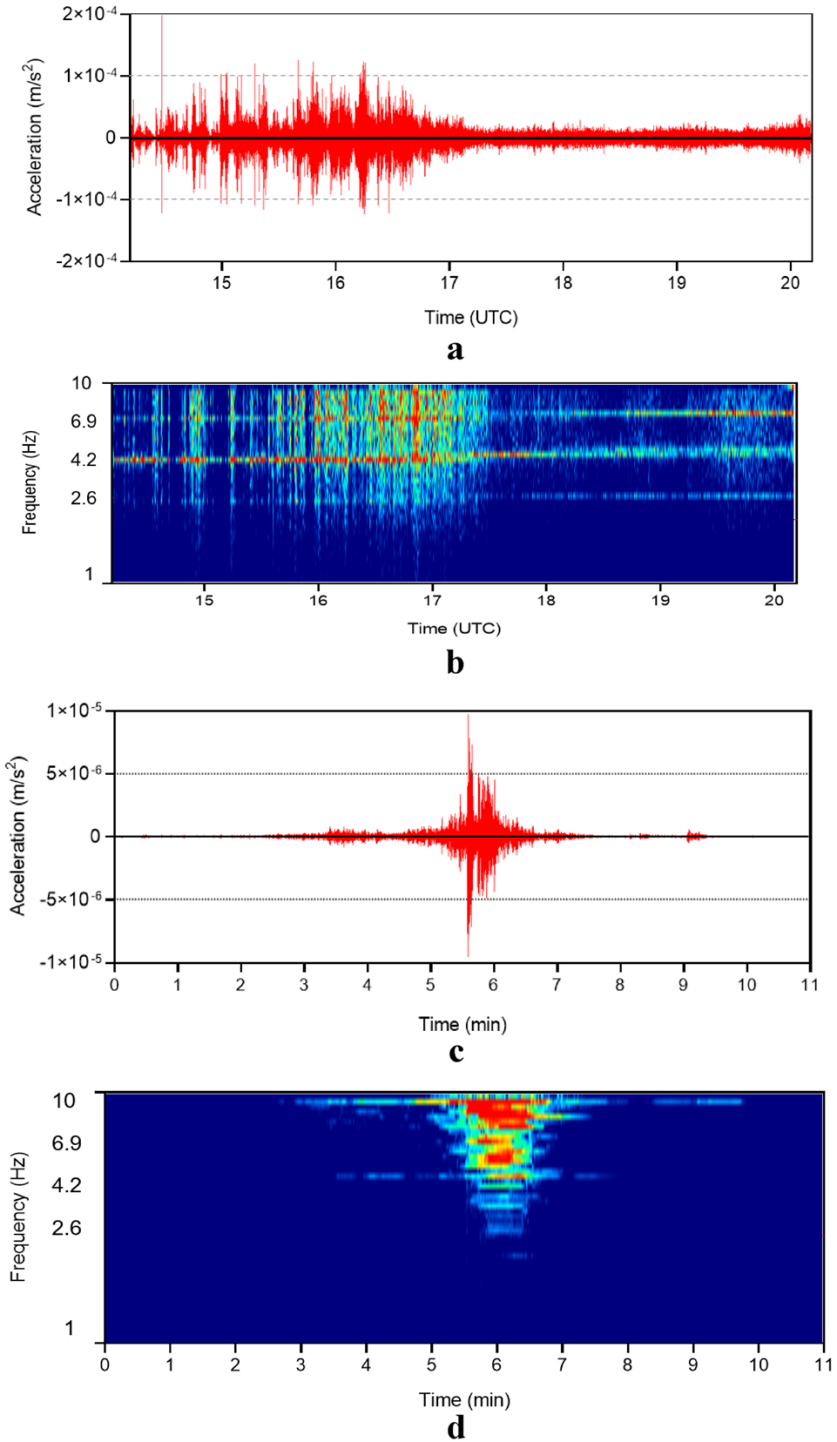
SEIS records. **(a)** Acceleration of V-orient of 2018/12/24 event. (**b)** Frequency content of V-orient of 2018/12/24 event. (**c)** Acceleration of U-orient of 2019/02/10 event. (**d)** Frequency content of U-orient of 2019/02/10 event.

The most effective approach to examining such records is after elimination of background ambient and wind noise. Figure 2a,b show over 6 h of calculated acceleration and frequency content recorded by SEIS through 24 December 2018 during a noisy sol^23,24^. Three frequencies of 2.6, 4.2 and 6.9 Hz continuously exist (Fig. 2b). This could have been related to excitation of the lander modes due to ambient or atmospheric disturbances. The calculated marsquake acceleration and frequency content of very broad band (VBB) sensor on February 10, 2019 were shown in Fig. 2c,d, respectively^23,24^. Interestingly, this record featured a relatively silent background. Considering analyzed Martian seismic events by Giardini et al. and using Eq. (5), the highest likely marsquake peak ground acceleration (PGA) was calculated 0.018 m/s^2^ (Table 2).

### Asteroid and meteoroid impact loads and effect

Because Mars has a thinner atmosphere and lower planet escape velocity than Earth (about 0.45), the probability of an asteroid hitting the surface of Mars is much higher than for Earth. Studies have shown that, on average, 200 asteroids having an average length of 1.5 m hit Mars annually.^7,25,26^ Eqs. (6 and 7) calculated the annual probability (${P}_{ht}$) of an average-sized asteroid hitting a settlement of the size of the Model-1 on Mars assuming a spherical shape for the asteroids as $4.32\times {10}^{-10}$. For a value of ${P}_{ht}$ (reliability index (β) of 6.13 and assuming the asteroid impact to be the only failure reason), this impact probability is very low. Generally, ${\beta }\approx 5.07$ is considered to be the reliability index for airborne elements^27^.

Meteoroids and micro-meteoroids also should be considered in the design of Martian structures. The thicker atmosphere of the Earth and its faster escape velocity (11,180 m/s) compared to Mars (5030 m/s) can prevent the passage of these bodies. By comparison, a meteoroid influx on Mars could be about four times that on Earth. Surprisingly, the thin atmosphere of Mars works as a shelter against meteoroid impacts^6,7,28^. However, particles with diameters of 60–1200 µm could reach the surface of Mars^29^. A density of 1000 kg/m^3^ has been considered for such particles^30^. Considering the characteristics of the planet, the terminal velocity for a particle of 125 µm in size with a roughly spherical shape has been calculated to be 9.07 m/s (Fig. 8). Because of the height of the Martian atmosphere, the terminal velocity will be equal to the impact velocity according to Eqs. (8–11)^31^. Even without consideration of air resistance force and buoyancy, the impact force and stress of the particle would be $3.78\times {10}^{-9}$ N and 0.30 Pa, respectively.

Because of the high influx, meteoroids would impact Martian structures. However, because of the pressure difference between the interior and exterior of the structure, structural sealing and permeability are of importance. This means that the amount of penetration due to the impact of such particles on Martian structures should be calculated. This amount was calculated using Eqs. (12–17) as being $2.62\times {10}^{-8}$ m for a particle (Fig. 9) with a calculated terminal velocity that impacts a block of concrete having a compressive strength of 50 MPa^32,33^. Even a particle of 1264 µm in diameter, which is less likely to impact the structure because of the very low influx, will have an impact penetration of about $5.50\times {10}^{-8}$ m.

### Martian structural load map

Table 1 presents the Martian structural load map for which a Martian structure should be designed to properly tolerate Martian conditions.

**Table 1:** Martian structural load map.

Load	Specification	Reason
Self-weight	As it is (not much as on the Earth)	Gravity acceleration equal to 3.71 m/s^2^
Pressure difference	1 atm (constant distributed force inside the structure)	Suitable pressure for human comfort
Wind	Relatively insignificant	Thin atmosphere and low atmospheric density of the planet
Marsquake	Negligible	Because nature characteristics of the planet like tectonic condition, depth and very low PGA
Asteroid impact	Negligible	Low influx (the annual probability of impact with a settlement is very low)
Meteoroids impact	Negligible	The penetration due to impact is not remarkable (low impact speed due to particle mass, buoyancy and drag force)

### Proposed models

**Figure 3:**
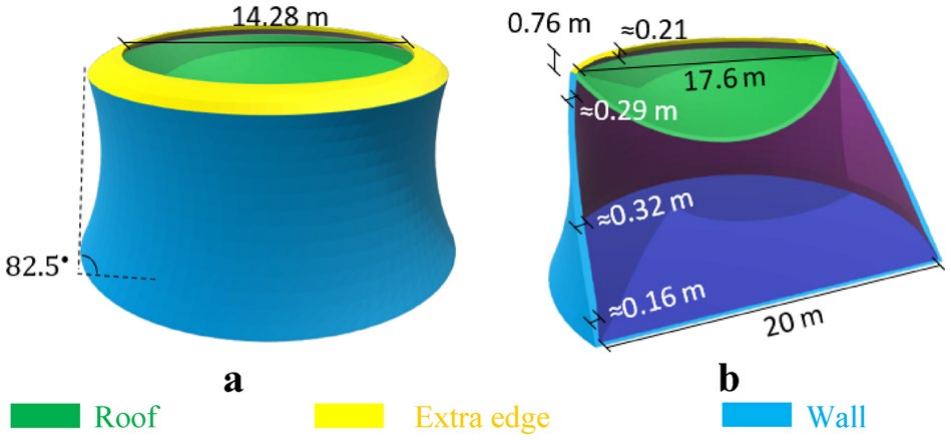
Proposed Martian structure, Model-1. **(a)** 3D view, (**b)** elevation view.

Figure 3a,b show Model-1 in which arches are used to minimize the tension stress of the structure (Fig. 10a,c and Eq. 18). The base radius and total height of the model are 10 m and 9.96 m, respectively. To accommodate architectural and structural limitations, h/b ratios of 0.12 and 0.20 have been considered for the walls and roof, respectively. An extra edge has been included to reduce and control the ring stresses in order to control concentrated stresses caused by roof deformation and tensile stress at the ring. This extra edge design behaves as compression under structural loads. Also, the ratio of the top perimeter to base perimeter was considered 0.88 for this model that provides walls with almost 82.5° and improve the behavior of the structure under applied Martian structural loads. Despite the very low structural wind pressure, the circular shape of the model provides an aerodynamic shape against wind loads.

**Figure 4:**
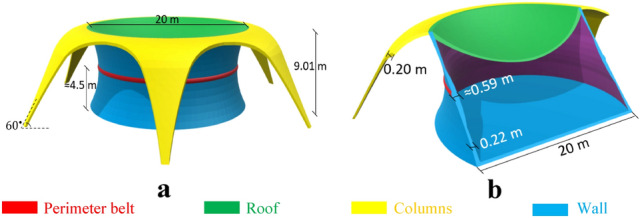
Proposed Martian structure, Model-2. **(a)** 3D view, (**b)** elevation view.

The total height and base radius of Model-2 are 9.01 m and 10.00 m, respectively (Fig. 4a,b). this model was designed with an h/b ratio of 0.155 and 0.17 for wall and roof, respectively. Six columns placed at an angle of 60° with a thickness of 0.20 m are used to control and transfer the ring stresses to the ground (the ring is surrounded by columns). The columns cause the wall behaves as the both end fixed arch. Thus, they behave as compression in the Y direction. Also, a perimeter was considered to control high stresses at almost the middle height of the structure.

**Figure 5:**
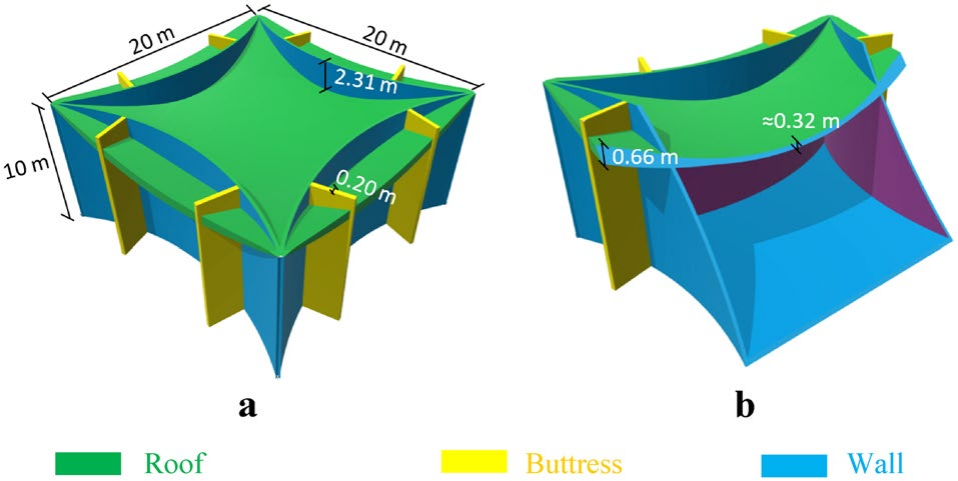
Proposed Martian structure, Model-3. (**a)** 3D view, (**b)** elevation view.

As Fig. 5a,b show, Model-3 was designed using four arches as walls with lengths of 20.0 m and a h/b ratio of 0.15. Two buttresses with thicknesses of 20 cm have been used on each side. The area and the maximum height of this model are 211.52 m^2^ and 10 m. respectively. A constant thickness of 20 cm has been considered for the walls. Table 3 presents the details of the models.

### Behavior of models

The maximum principal logarithmic strain (MPLS) and the plastic strain magnitude (PSM) of the models under 0% (only gravity load), 50% (50,662.50 Pa), and 100% (101,325 Pa) of atmospheric pressure as internal pressure and the gravity load, at the same time (Table 1 for determinative loads) are shown in Table 4. Under 100% of atmospheric pressure, only the proposed models show elastic behavior. For example, under 50% of atmospheric pressure, Model-1 shows 1.98 MPa maximum tensile stress which is almost 50% and 21% fewer than the Kozicki and Kozicka^12^ and Cesaretti et al.^13^ models, respectively. For plastic strain which is related to the damage and cracks, under 50% of atmospheric pressure, Model-1 shows 54.63% and 17.22% fewer MPLS than the concrete structural forms of Kozicki and Kozicka^12^ and Cesaretti et al.^13^, respectively (Model-1 shows no plastic strain). The proposed models are the only ones that show complete elastic behavior under 1 atm of internal pressure.

The high plastic strain affects strongly the air permeability coefficient of the concrete^34^. Thus, to provide an acceptable air seal, the concrete of the Martian structures should record no plastic strain under structural loads. On Mars, air leakage will cause freezing, expansion, and ultimately, crack propagation. Hence, under 100% of atmospheric loading, even the concrete structural form of the Cesaretti et al. proved unacceptable^13^.

**Figure 6:**
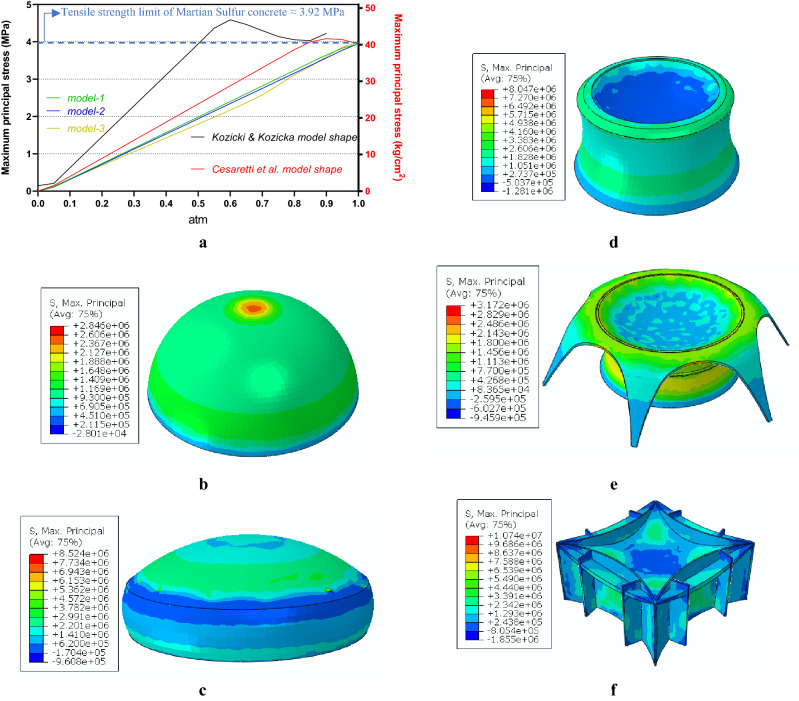
MPS of models. **(a)** Under gravity load and 0–1 atm of internal atmospheric pressure. (**b)** Cesaretti et al. concrete structural form under 50% of internal atmospheric pressure. (**c)** Kozicki and Kozicka concrete structural form under 50% of internal atmospheric pressure. (**d)** Model-1 under 50% of internal atmospheric pressure. (**e)** Model-2 under 50% of internal atmospheric pressure. (**f)** Model-3 under 50% of internal atmospheric pressure.

Figure 6a shows the maximum principal stress (MPS) of models under gravity loading with different amounts of internal pressure. The diagrams show that under 50% and 85% of atmospheric pressure, the Cesaretti et al.^13^ and the Kozicki and Kozicka^12^ concrete structural forms showed non-linear behavior. which is important for permeability Furthermore, the Kozicki and Kozicka concrete structural form failed under 60% of atmospheric pressure^12^. On the other hand, the proposed models showed remarkably less MPS during loading with much less slope as well as linear (elastic) behavior. For example, at 50% of atmospheric pressure, the MPS of the Model-1 was 0.35 and 1.92 MPa less than for the Cesaretti et al.^13^ and Kozicki and Kozicka^12^ concrete structural forms, respectively.

The MPS contours of the models under 50% of atmospheric pressure are shown in Fig. 6b– f. It is evident that there is little stress concentration and better stress distribution in Model-1 and Model-2, meaning they are more efficient forms compared to the other models. These two forms could be used for Martian settlements without the need to import structural elements from Earth.

In-place Martian concretes and binders such as plaster of Paris (POP), ordinary Portland cement (OPC), alkali-activated cement, sulfur concrete, Geopolymer cement, and Mg-based cement show brittle behavior under loads with very low tensile strength and relatively suitable compressive strength. The proposed structures (chiefly Model-1) were designed to minimize the tensile stress and maximize compressive stress to enable the use of in-place concretes to show appropriate and linear behavior under Martian structural loads using only Martian concretes. Thus, these structural systems can be constructed using concretes other than sulfur concrete.

### Concrete volume for construction

**Figure 7:**
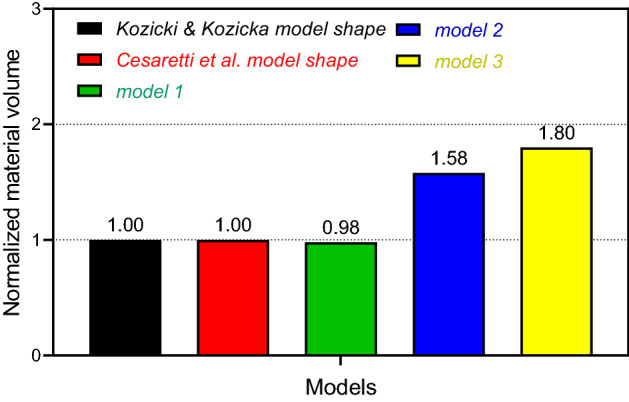
Comparison of required construction material volume of models.

The energy available is a concern on Mars. Less concrete for construction reduces required energy. Because of the distance of Mars from the Sun (negative effect) and the thin and clear atmosphere (positive effect), the amount of solar energy which reaches the surface of Mars is around 41% lower than the Earth (Fig. 12). Figure 7 shows the volume of concrete required by the models normalized to the volumes of the Kozicki and Kozicka concrete structural form^12^.

Model-1 requires about 2% less concrete material during construction than the models of Cesaretti et al.^13^ and Kozicki and Kozicka^12^. This means it requires less energy for mining sulfur from the regolith, melting sulfur, finding suitable aggregate and the 3D printing process. In addition, there will be lower costs because the import structural elements from Earth is not required, which will mean a significant reduction in cost, energy, and time spent, which are major consideration for Martian colonization. Another advantage of this model is the possibility of optimization of the weight of the roof (due to gravity) and the lifting force (due to 1 atm of pressure).

## Conclusions

The need to explore an unknown environment is an inherent characteristic of human beings. Multiplanetary life will happen for future generations. It could occur relatively soon and Mars will likely be the first destination. Mars colonization requires low-cost, low-energy, reliable and stable structures for its first settlement. Our study addressed these issues by proposing innovative structural forms. Investigation and calculation of the lateral loads of wind and marsquakes using recorded data from the landers (Viking 1 and 2 and InSight), gravity, internal pressure, and role of asteroid and micro-meteoroids indicate that the self-weight load from gravity and the pressure difference between the interior and exterior are crucial loads for Martian structures. The proposed models show completely elastic behavior under Martian structural loads, which is essential for the permeability of Martian structures. While the concrete structural forms of previous studies showed plastic behavior under 50% to 100% of internal atmospheric pressure, Model-1 requires less concrete and less construction energy. Besides the better structural behavior and less concrete requirement for construction, Model-1 has a simpler geometry than Model-2 and Model-3 that makes construction and printing process easier. The results showed that the proposed models eliminate the need for costly importation of terrestrial structural units such as inflatable elements from the Earth. Additionally, because of the similar behavior of the Martian in-place concretes, very limited tensile strength and almost suitable compressive strength, and linear behavior of the proposed Martian structures it is possible to use these structural forms for different kinds of in-place Martian concretes such as POP, OPC, alkali-activated cement, sulfur concrete, Geopolymer cement, Mg-based cement, etc.

Of note, this study only proposed a new generation of Martian structural forms. Other challenges that should be considered for a Martian building such as radiation shield, required thermal energy, internal human-friendly design, mechanical equipment, etc. should be studied by relevant experts.

## Methods

### Martian structural loads

The general structural stability, geometric combability, and adequation of a structure in the short- and long-term should be assessed using valid and appropriate analysis methods. Although these loads can be categorized as being live or dead loads, in this study, the structural loads were assessed as being either vertical or lateral loads^35^.

### Wind speed

Equations (1–3) were used for Earth conditions, wind stagnation pressure and velocity pressure for a structure the size of Model-1^21^:

1 $$\varphi =0.613{v}^{2}=411.20\frac{\mathrm{N}}{{\mathrm{m}}^{2}}$$

2 $${q}_{z}=\varphi {K}_{Z}{K}_{Zt}{K}_{d}=342.52\frac{\mathrm{N}}{{\mathrm{m}}^{2}}$$

3 $$F={q}_{z}A=59917.02 \mathrm{N}$$

where $\varphi$ is the wind stagnation pressure, $v$ is the maximum wind velocity, ${q}_{z}$ is the velocity pressure at height *z*, ${K}_{Z}$ is the velocity pressure exposure coefficient (type c: open terrain with scattered obstructions), ${K}_{Zt}$ is the topographic factor (flat terrain = 1), ${K}_{d}$ is the wind direction factor, $F$ is the wind force and $A$ is the structure area.

In climatology, wind is the motion of gases relative to the surface. The wind force or pressure depends on the air density (ρ) or its mass per volume. Therefore, a heavier atmosphere applies more wind pressure on the structure. The atmospheric conditions of Mars are completely different from that of Earth. The fluid dynamic pressure can be calculated using Eq. (4) by considering Mars air density as^36,37^:

4 $$\varphi =0.5\rho {v}^{2}$$

### Marsquake

Tectonic plates are the main causes of shallow strong ground motions. On Earth, shallow ground motions comprise the most numerous earthquakes at depths of 0–70 km. Destructive earthquakes usually occur at depths of less than 15 km on the crust. The closer to the surface that an earthquake occurs, the greater the chance for destruction (higher PGA). However, there is no clear evidence for plate tectonics on any planet except Earth. This is no exception for Mars. Some researchers have proposed that the crust of Mars could have been seismically active millions of years ago^38–40^. However, all the remarkable (low frequency) marsquakes that have been recorded (24 numbers) originated in the mantle^22^. Of note, by considering the size of the two planets, that the crust of Mars has an average depth of 50 km, which is about three times that of Earth. Therefore, marsquake waves should travel for longer distances (from the mantel to the surface) in comparison with shallow earthquakes and this would strongly dampen the motion components. A magnitude 4 Richter marsquake would be much less dangerous than a 4 Richter earthquake, which is not sensible for human.

Studies have shown that Mars has an internal structure that is similar to that of Earth and terrestrial-like activity^8,38,40^. Assuming wave propagation that is similar to that of the Earth, empirical predictive Eq. (5) can be used to calculate the highest likely PGA of a marsquake in accordance with the largest events analyzed by Giardini et al.^22,41,42^. Table 2 shows that the calculations were based on the worst-case scenario or the highest likely PGA. In this regard, the distance to the epicenter and focal depth were assumed to be 0 km and 50 km, respectively. Marsquakes probably occur a greater assumed epicenter distance and focal depth, which would decrease the PGA. Table 2 shows the calculated highest likely PGA of the largest seismic events on Mars:

5 $${(\mathrm{log}PGA)}_{\frac{\mathrm{cm}}{{\mathrm{s}}^{2}}}=0.67+0.43M-1.08\mathrm{log}{\left({R}^{2}+{h}^{2}\right)}^{0.5}\pm0.32$$

where $M$ is the magnitude (Richter), $R$ is the distance to the epicenter (here, 0 km), $h$ is the focal depth (assumed 50 km) and 0.32 is the standard error.

**Table 2:** Highest likely PGA of some largest marsquake events.

Event sol	Date	Magnitude *M*_*W*_	Calculated PGA-σ (m/s^2^)	Calculated PGA (m/s^2^)	Calculated PGA + σ (m/s^2^)
325	2019/10/26	3.7	0.008	0.018	0.03
173	2019/05/23	3.6	0.008	0.016	0.03
154	2019/05/04	3.5	0.007	0.015	0.03
226	2019/07/15	3.2	0.005	0.011	0.02
183	2019/06/03	3.1	0.005	0.010	0.02
189	2019/06/09	3.0	0.004	0.009	0.01
234	2019/07/23	2.8	0.003	0.007	0.01

### Asteroid and meteoroid impacts

Equations (6 and 7) below show the calculated annually impact probability of asteroids on a structure the size of Model-1:

6 $${P}_{h}=\frac{a(h)}{{A}_{m}}\times \mathrm{\alpha }=2.16\times {10}^{-12}$$

7 $${P}_{ht}=\sum_{1}^{200}{P}_{h}=4.32\times {10}^{-10}$$

where $a(h)$ is the impact area (average-sized asteroid area), ${A}_{m}$ is the total area of Mars, $\mathrm{\alpha }$ is the ratio of settlement area to asteroid area, ${P}_{h}$ is the annual probability that an average asteroid will hit a settlement the size of Model-1 and ${P}_{ht}$ is the total annual probability that any of 200 asteroids will hit a settlement the size of Model-1.

### Meteoroid impact

**Figure 8:**
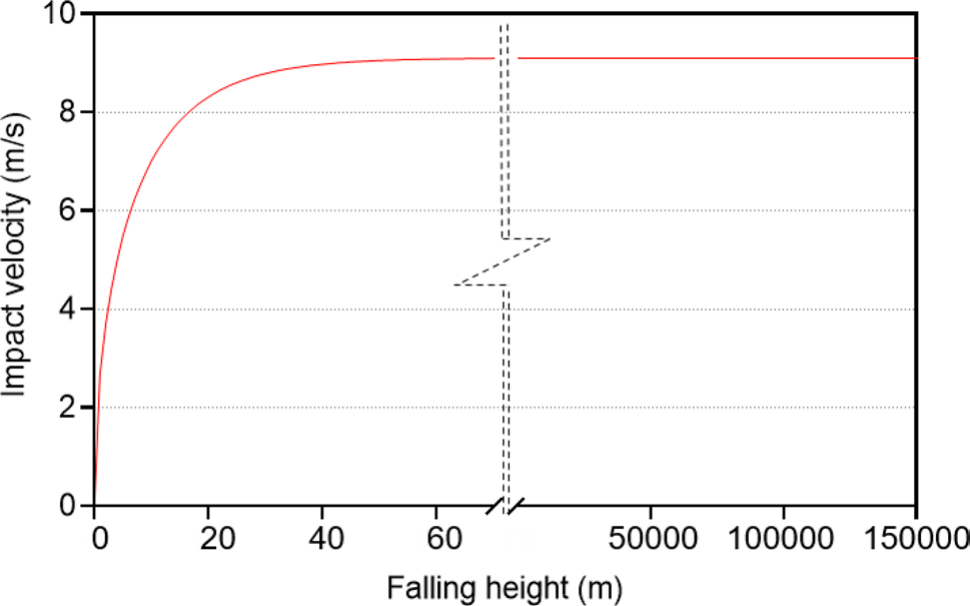
Terminal velocity. Terminal velocity of a meteoroid particle in size of 125 µm through the Mars atmosphere height.

Figure 8 shows the terminal and impact velocity through the height, as calculated in Eqs. (8–11):

8 $${v}_{t}=\sqrt{\frac{2m{g}_{m}}{C\rho {A}_{p}}}$$

where $m$ is the particle mass, ${g}_{m}$ is the Mars gravity acceleration, $C$ is the drag coefficient, ρ is the density of fluid and ${A}_{p}$ is the area of the particle:

9 $$\tau =\frac{{v}_{t}}{{g}_{m}}$$

10 $${t}_{impact}=\tau {\mathrm{cosh}}^{-1}\left[exp\left[\frac{{y}_{peak}}{{v}_{t}\tau }\right]\right]$$

11 $${v}_{impact}={v}_{t}\sqrt{1-exp\left[\frac{-2g{y}_{peak}}{{v}_{t}^{2}}\right]}$$

where $\tau$ is the characteristic time, ${t}_{impact}$ is the impact time, ${y}_{peak}$ is the falling height and ${v}_{impact}$ is the impact velocity.

**Figure 9:**
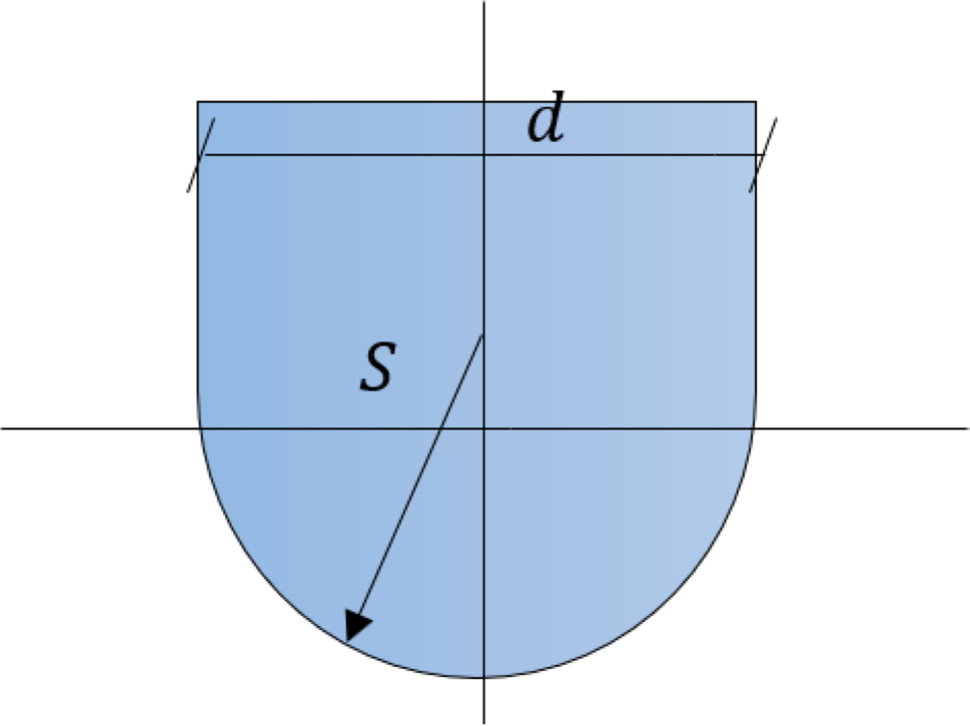
Blunt-nose penetrator. Assumed shape of meteoroid particles impacting Martian structures and surface.

Equations (12–17) were used to calculate the penetration distance with the assumption of a blunt-nose shape for a meteoroid (Fig. 9)^24,32^:

12 $${D}_{1}=0.3SN{\left(\frac{W}{A}\right)}^{0.7}Ln(1+2{V}^{2}\times {10}^{-5})$$

13 $${D}_{2}={K}_{h}{D}_{1}$$

14 $${K}_{h}=0.4{\left(W\right)}^{0.15}$$

15 $$N=\frac{0.09\left({L}_{n}+{L}_{n}^{^{\prime}}\right)}{d}+0.56$$

16 $$S=0.85{K}_{e}\left(11-P\right){\left({t}_{c}{T}_{c}\right)}^{-0.06}\left(\frac{5000}{{{f}^{^{\prime}}}_{c}}\right)$$

17 $${K}_{e}=(\frac{F}{{W}_{1}})$$

where ${D}_{1}$ is the penetration distance (ft), ${D}_{2}$ is the modified penetration distance (effect of low weight and high speed), $S$ is the s-number (dimensionless), $N$ is the nose performance coefficient (dimensionless), $W$ is the weight of the penetrator (lbs.), $A$ is the cross-sectional area (in^2^), $V$ is the impact velocity (fps), ${K}_{h}$ is the modification coefficient (for a low weight penetrator), ${L}_{n}$ is the length of the penetrator nose (in), ${L}_{n}^{^{\prime}}$ is the actual nose length after the original (not blunted) length is reduced by the blunting, $d$ is the penetrator diameter (in), ${K}_{e}$ is the correction for edge effects in the concrete target, $P$ is the volumetric percentage of rebar (0% for non-reinforced concrete), ${t}_{c}$ is the concrete curing time (if ${t}_{c}>1$, then set ${t}_{c}=1$), ${T}_{c}$ is the thickness of the target in penetrator diameters, ${{f}^{^{\prime}}}_{c}$ is the unconfined compressive strength at test time (psi), $F$ is equal to 20 for reinforced concrete and 30 for no reinforcement and ${W}_{1}$ is the target width in penetrator calibers (if W1 > F, then ${K}_{e}$ = 1).

### Arches

**Figure 10:**
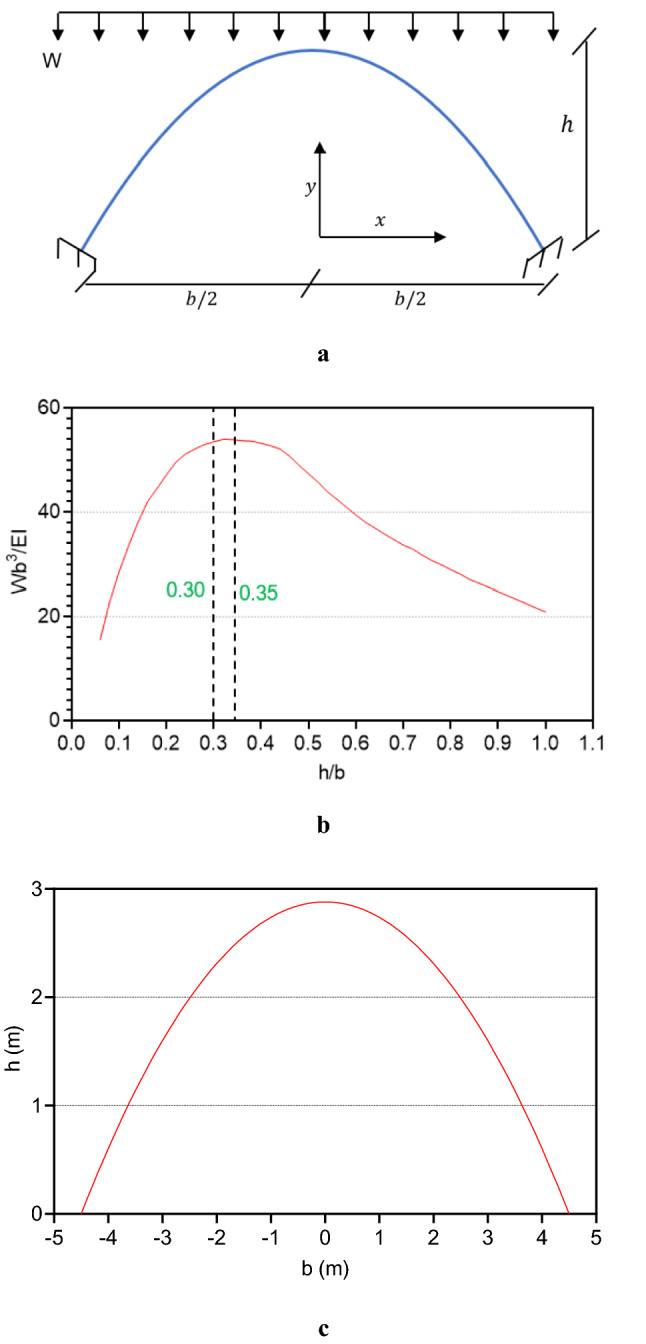
Parabolic-symmetric fixed arch. **(a)** Arch scheme under uniform load. (**b)** Buckling load diagram for height to span-length ratios. (**c)** Optimum parabolic arch for h/b = 0.32 and span length of 9 m.

Arches have the ability to convert any vertical load to a compressive load along the arch direction^43^. Because of the low tensile strength and relatively high compressive strength of available Martian binders and concretes, arch elements are suitable forms for Martian structures. The current study proposed and analyzed suitable Martian structure forms using rotated arches. Figure 10a shows a 2D parabolic symmetric fixed arch under a uniform load of W (1 atm of internal pressure uniformly applied in the interior of the structure). The buckling vertical load ratio of height-to-span-length diagram of the intended arch is shown in Fig. 10b. It can be seen that the optimum performance of this kind of arch is in the range of 0.30 ⩽ h/b ⩽ 0.35^44,45^. The optimum arch shape can be calculated according to Eq. (18)^43,45^. Using this equation and Fig. 10b, the optimum shape of a symmetric parabolic arch under uniform loading with a height of 2.88 m and a span length of 9 m (h/b = 0.32) was calculated and is shown in Fig. 10c.

18 $$y=h(1-\frac{4{x}^{2}}{{b}^{2}})$$

where $h$ is the height of the symmetric arch, $x,y$ are the cartesian positions of the arch and $b$ is the horizontal span length.

### Material property

The chosen concrete should be appropriate for 3D printing and durable under Martian atmospheric conditions. Mars is a sulfur-rich (S) planet. Observations show that on average, 6.8 wt% of Martian dust (2.7 wt% S) and 6.2 wt% of the soil (2.48 wt% S) are composed of SO_3_^46,47^. This makes sulfur concrete a good choice for Martian construction; thus, the current study used material properties of sulfur concrete to analyze.

**Figure 11:**
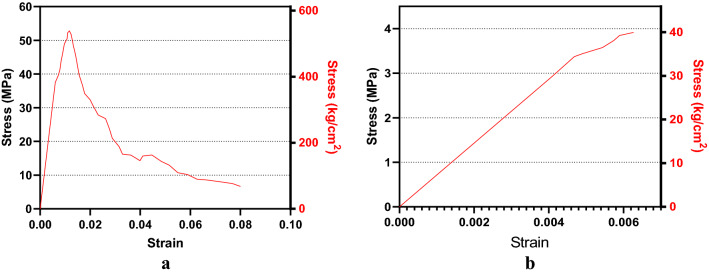
Simulated Martian concrete average stress–strain behavior. **(a)** Compression. (**b)** Tension.

Sulfur concrete is waterless, can be used in 3D printers and workable in the temperatures and atmosphere of Mars. Additionally, its production requires less energy than other binders, including Martian OPC and basalt concrete. Studies have shown that the highly varied temperature of Mars from day to night and season to season does not affect the durability of sulfur concrete (intra-stress envelope)^14,33,48^. A low temperature will actually increase curing of this type of concrete. Furthermore, sulfur is in a rhombic phase in the Martian environment and atmospheric conditions. The current study used the average stress–strain curve for sulfur concrete from Wendner et al.^33^. The average strain–stress curves of the intended Martian sulfur concrete were shown in Fig. 11a,b, respectively.

It should be noted that in the presence of moisture, sulfur may produce toxic gases. To prevent this, an appropriate thin layer of plaster is proposed for the internal wall and roof. This layer is not a structural component and would tolerate no structural loads. It only decreases the permeability and prevents the production of toxic gases.

### Comparison of models

The behavior of the proposed models was compared to the concrete structural forms from Cesaretti et al.^13^ and the residual dome (RD) of Kozicki and Kozicka^12^ which also have been built using sulfur concrete. For the purposes of comparison, the volumes of all five models were chosen to be almost similar to one another. The volume of the RD dome from the Kozicki and Kozicka model was considered as the reference volume. The models showed a maximum volume difference of 2.08%. Table 3 shows the specifications of the models.

**Table 3:** Model specifications.

Model	Radius (m)	Area (m^2^)	Total height (m)	Volume (m^3^)
Cesaretti et al.	10.00	314.15	5.60	1721.38
Kozicki & Kozicka	10.00	314.15	7.29	1705.26
Model-1	10.00	314.15	9.20	1713.70
Model-2	10.00	314.15	9.00	1741.22
Model-3	–	211.52	10.00	1703.63

Abaqus software was used to perform non-linear dynamic FE implicit analysis. This software has a good ability to analyze models with concrete-like behavior like this study including initial damage, damage evolution, strength and stiffness deterioration^49,50^.

The strain parameter is more sensitive than displacement as the structural damage control criteria^51^. The plastic magnitude strain (PMS), the MPLS and the MPS were considered as the control criteria. The concrete damage plasticity model was chosen for modeling sulfur concrete behavior based on the sulfur concrete stress–strain behavior^50^. The analysis method was the implicit Hilber-Hughes-Taylor method with a full Newton–Raphson solution for greater accuracy^52^. Table 4 shows the MPLS and PMS of the models under gravity loads and different amounts of atmospheric internal pressure.

**Table 4:** MPLS and PSM of models under gravity loading at 0%, 50% and 100% internal atmospheric pressure.

Model	0% atm		50% atm (50,662.50 Pa)		100% atm (101,325 Pa)	
	MPLS	PSM	MPLS	PSM	MPLS	PSM
Cesaretti et al.	8.00 × 10^–07^	0	3.31 × 10^–05^	0	8.42 × 10^–05^	3.80 × 10^–05^
Kozicki & Kozicka	2.76 × 10^–06^	0	6.04 × 10^–05^	0	Failed	Failed
Model-1	1.06 × 10^–06^	0	2.81 × 10^–05^	0	5.77 × 10^–05^	0
Model-2	1.54 × 10^–06^	0	2.75 × 10^–05^	0	5.67 × 10^–05^	0
Model-3	2.13 × 10^–06^	0	2.82 × 10^–05^	0	6.20 × 10^–05^	0

### Importance of energy on Mars

Energy and time are some of the greatest concerns in deep space exploration and for Martian colonization^53^. Outside the atmosphere of Earth, the amount of the power per unit area (W/m^2^) received from the Sun at noon is about 1300 W/m^2^. On a clear sunny day (spring or summer), the maximum solar irradiance that reaches the Earth’s surface at noon is 950–1000 W/m^2^. This value is 430–590 W/m^2^ for a sunny day on Mars; therefore, the amount of solar energy is much lower.

**Figure 12:**
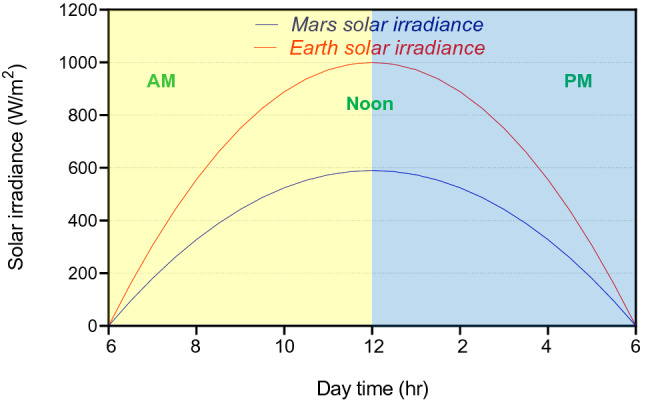
Comparison of approximate solar irradiance on Earth and Mars on a sunny summer day.

The concrete volume required for construction is determinative. Figure 12 shows the approximate Mars and Earth solar irradiance on a sunny day using a parabolic equation. Other methods of producing energy exist on Mars, such as geothermal and cosmic energy^54,55^. However, many will not be available in the early stages of colonization.

## Data Availability

The data that support the findings of this study or the raw data used during the current study are available on reasonable request from the corresponding author.
